# The status and future of emergency care in the Republic of Kenya

**DOI:** 10.1016/j.afjem.2021.11.003

**Published:** 2022-01-12

**Authors:** J. Austin Lee, Grace Wanjiku, Naomi Nduku, Adam R. Aluisio, Ramu Kharel, John Tabu Simiyu, Benjamin W. Wachira

**Affiliations:** a Department of Emergency Medicine, Brown University Warren Alpert Medical School, United States of America; b Presbyterian Church of East Africa, Chogoria Mission Hospital, Kenya; c Moi University College of Health Sciences, Kenya; d The Aga Khan University, Nairobi, Kenya

**Keywords:** Kenya, Emergency care, Devolution, Universal health coverage, Medical education

## Abstract

Kenya is a rapidly developing country with a growing economy and evolving health care system. In the decade since the last publication on the state of emergency care in Kenya, significant developments have occurred in the country's approach to emergency care. Importantly, the country decentralized most health care functions to county governments in 2013. Despite the triple burden of traumatic, communicable, and non-communicable diseases, the structure of the health care system in the Republic of Kenya is evolving to adapt to the important role for the care of emergent medical conditions. This report provides a ten-year interval update on the current state of the development of emergency medical care and training in Kenya, and looks ahead towards areas for growth and development. Of particular focus is the role emergency care plays in Universal Health Coverage, and adapting to challenges from the devolution of health care.

## African relevance

• Kenya is a rapidly developing middle-income African country with an evolving emergency care landscape
• Emergency medicine is a recognized specialty in Kenya, and there has been increasing development of EM education
• The Kenyan government has shifted a number of domains, including healthcare, to the county level (devolution) which has led to unique challenges
• To attain Universal Health Coverage, improvements to the quality and accessibility of emergency care will be necessary
• Challenges to strengthening emergency care in Kenya are unique but may be generalizable to other African countries

## Introduction

Emergency health conditions represent a high burden of disease and improved care provides an opportunity for a healthier Kenya 1., 2.. Emergent conditions account for substantial morbidity and mortality in low and middle-income countries (LMICs) yet care for these conditions has been a neglected area of health systems in much of the world 3., 4., 5.. The last decade has seen increased focus and development of emergency medicine in the Republic of Kenya. Since the last publication regarding the state of emergency medical care in Kenya one decade ago, there have been several developments to the country's approach to emergency medical care [1]. This article provides an update regarding advances to emergency care systems, education, and policy in Kenya, while also proposing advancement targets for the future of emergency care in the country.

## Republic of Kenya

Kenya is a country located in East Africa covering an area of around 580,000 km^2^ and is divided into 47 counties [6]. The counties can be grouped into six regional economic blocs; comprised of three to fourteen counties, these blocs have formed among politically and economically similar neighbors [7]. The country has a population of 47.6 million, and 69% of the population lives in rural areas 8., 9.. The Kenyan government underwent major restructuring in 2013 (following the 2010 constitution amendment), when many services transitioned from the central government to the 47 semi-autonomous county governments [10]. The process of devolution moved a number of government health care responsibilities, including those for local health facilities, pharmacies, ambulance services, and the promotion of primary health care, to county governments [11].

## Structure of Kenyan health system

Today, the Kenyan health system is facing a triple burden of disease (communicable disease, non-communicable disease, and trauma) [2]. Progress in recent years has led to a steady decrease in the incidence of communicable diseases (particularly, HIV/AIDS, diarrheal disease, respiratory infections, and malaria), which has been attributed to disease-specific programming and the Kenya Expanded Programme on Immunization [12].

**Fig. 1:**
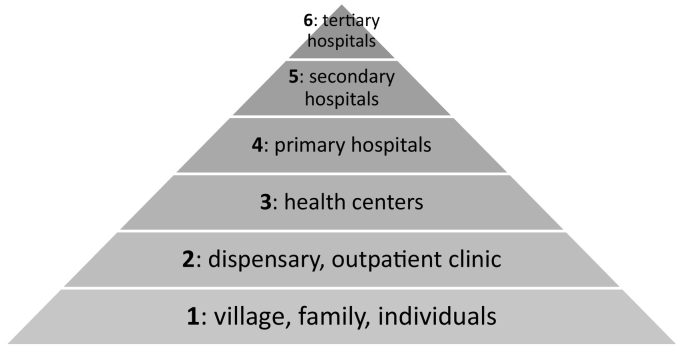
Levels of the Kenyan health system.

The provision of healthcare services in Kenya is through a network of 13,058 health facilities spread across the country [13]. Most facilities are public (47%) or private (42%), while faith-based organizations (8%) and non-governmental organizations (3%) comprise the rest [13]. Healthcare in Kenya is delivered in a hierarchical system involving 6 levels as outlined in the 2017 Health Act [14]. The national government plays a leading role in developing policies, laws, procedures, and programs in consultation with the semi-autonomous county governments, health stakeholders, and the public [14]. Devolution of the Kenyan health system shifted responsibility to county governments for managing level one up to level five health facilities (Fig. 1). The national government manages all level 6 facilities: Kenyatta National Hospital, Moi Teaching and Referral Hospital, Kenyatta University Teaching Referral and Research Hospital, Mathari Hospital, and the National Spinal Injury Referral Hospital [11].

(Adapted 11., 14.)

## Kenyan health system financing

Kenya has oriented its health care goals towards the provision of Universal Health Care (UHC) to all citizens 12., 15.. UHC is based on the goal that all individuals have access to any needed health services, when and where they are needed, without creating an undue financial burden [16]. Health care financing towards UHC in Kenya comes from a complement of taxes, out-of-pocket payments, private insurance, the National Hospital Insurance Fund (NHIF), and donor funds (which are generally narrow and programmatic) [17]. Current funding for emergency care relies heavily on individual's out of pocket payments, with contributions from NHIF or private insurance available to covered individuals. While some believe the Kenyan government is working towards a single-payer system, challenges will include integrating national and county contributions, coverage of individuals with informal income sources, and responsibility for the poor and vulnerable [18]. Recent efforts have focused on expanding and reforming the NHIF services to reduce costs and improve the quality and availability of covered services 15., 17.. Additionally, the 2017 Health Act established the Emergency Medical Treatment Fund to set aside funds for supplementary financing of unplanned emergencies such as a natural disaster or epidemic outbreaks [14].

## Emergency care and universal health care

The availability of adequate, 24-hour care for unscheduled acute health concerns is a cornerstone of the provision of universal health care, a fact that has been emphasized by the World Health Organization (WHO) in the World Health Assembly resolution 72.16 [19]. As noted, “emergency care is an integrated platform for delivering accessible, quality and time-sensitive health care services for acute illness and injury across the life course” [19]. The WHO emergency care systems framework has been developed to support health systems (from the local to national level) in developing and improving systems for emergency care. This framework emphasizes the importance of integrated emergency care systems, from the prehospital setting through to tertiary care receiving centers [20].

The Kenyan framework for the care of emergent conditions is built on a robust structure that is set forth in national law [21]. The Kenyan Constitution (in 2010) codified in the Bill of Rights that all Kenyan citizens “shall not be denied emergency medical treatment.” [22] Furthermore, the Health Act of 2017 defines a medical emergency as one that poses an “immediate risk to life or health of a person or has potential for deterioration”, and emergency treatment is that which is “necessary immediate health care that must be administered to prevent death or worsening of a medical situation” [14].

## Kenyan health education

Medical education in Kenya is based at one of 11 approved universities and six years of undergraduate medical education are split between pre-clinical work, and clinical rotations [23]. Medical school graduates then disseminate across Kenya to fulfill a 12-month internship where individuals rotate through internal medicine, surgery, pediatrics, obstetrics and gynecology, and psychiatry and community mental health 24., 25., 26.. Advanced training, in the form of certificate and diploma programs, as well as Masters of Medicine (MMed) programs are available in a number of specialties and further sub-specialty training is also available in Kenya.

Clinical officers complete a three year diploma program (or four year Bachelor's degree program), followed by a one year clinical practice internship; if desired, clinical officers can pursue advanced specialty training [27]. Education pathways for nurses range from two and a half years (basic diploma) to three and a half years (registered nurse) to four years with a subsequent one year internship (bachelor's degree) [28]. There are two levels of emergency medical service (EMS) providers (Emergency Medical Technician and Paramedics) and the education and training background of EMS providers is quite variable and poorly defined [29]. Existing EMS educational programs (through the Kenya Red Cross Training School or the Kenya Council of Emergency Medical Technicians) offer a certificate after 9 months of study for emergency medical technicians and a diploma after 12 months for paramedics 29., 30..

## State of emergency care in Kenya

Devolution has shifted emergency care from a one government, one health system approach to what is effectively 47 distinct county-based approaches. As most healthcare in Kenya is provided outside of the highest level six health facilities, each county government is responsible for building and maintaining an emergency care system. While devolution can lend the opportunity for tailored local solutions, it can also lead to fractured and less-coordinated approaches to care. Unfortunately, many geographic regions and levels of care within the Kenyan health system are ill-prepared to care for emergent conditions [31].

Prehospital emergency medical services are an integral piece of any emergency care system. Currently, due in large part to disparate county-based approaches, the Kenyan government cannot estimate the total number or location of ambulances across the country [32]. There is clearly a need for approaches and solutions that address the challenges of standardizing and coordinating care across 47 distinct EMS services. There is not a single national emergency response phone call line, though individual counties may have numbers available for emergency health, police, or fire services.

**Fig. 2:**
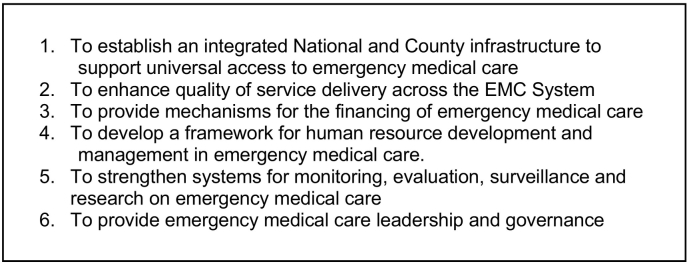
Objectives for the Kenya emergency medical care strategy (2020–2025) [32] and the Kenya emergency medical care policy (2020–2030) [33].

As the Kenyan Ministry of Health has recently noted: “despite the guarantee of the right to emergency medical treatment as defined in the Constitution of Kenya (2010), the Health Act (2017) and several strategic plans referencing the need for Emergency Medical Care Systems in Kenya there has been very little progress in actually implementing these interventions” [33]. In response, the Ministry of Health has recently prioritized emergency medical care across the continuum, with particular attention to the prehospital setting. The recently released Kenya Emergency Medical Care Strategy (2020–2025) and Kenya Emergency Medical Care Policy (2020−2030) are aimed at integrating efforts and improving the quality of emergency medical care, under the umbrella of providing UHC (Fig. 2) 32., 33..

## Emergency medicine as a specialty in Kenya

Emergency medicine was recognized (gazetted) as a distinct medical specialty in Kenya in 2017, and current regulations require a minimum duration of three years of postgraduate training with further two years of supervised practice [34]. There are currently three subspecialties available to Kenyans (pediatric emergency medicine, trauma surgery, and medical toxicology), which require six months of specialized training and a further one year of supervised practice [34].

## Emergency medicine education and training in Kenya

Within the Republic of Kenya, a diverse suite of educational and training efforts for emergency medicine are ongoing or under development, spanning from short courses to advanced post-graduate degree-granting programs.

## Focused training opportunities

A variety of short courses and certificate programs have been rolled out in Kenya, including courses that have been developed and piloted in other countries, as well as others that were developed within Kenya. These include the WHO Basic Emergency Care Course (with the related follow-on training-of trainers course), the WHO Emergency Triage Assessment and Treatment, and The Emergency Care Course developed by the Emergency Medicine Kenya Foundation 35., 36., 37.. A number of short courses have also aimed to fill educational gaps around trauma with work using the American College of Surgeons Trauma Evaluation and Management (TEAM) course, as well as the formal and modified Advanced Trauma Life Support (ATLS) courses 24., 25..

## Longitudinal training opportunities

The most robust training program currently operating is at the Aga Khan University, Nairobi where a cohort of five medical officers undergo an 18-month program with the end goal of qualifying for the South African Diploma in Primary Emergency Care [38]. An effort to develop an integrated Family And Emergency Medicine Residency Program for physicians is based at Sagam Community Hospital and is affiliated with Maseno University School of Medicine 39., 40.. The Kenya Medical Training College currently offers an 18-month program, the Higher Diploma in Emergency and Critical Care Medicine, which is available to both clinical officers and medical officers [41]. A one year program has been available since 2015 at the Kijabe Hospital, titled the Emergency and Critical Care Clinical Officers Diploma Program, which makes participants eligible to sit for the Higher Diploma in Emergency and Critical Care for Clinical Officers [42]. The Pediatric Emergency and Critical Care Kenya fellowship program is a two year program for pediatricians based at both Kenyatta National Hospital (in collaboration with the University of Nairobi) and Kijabe Hospital.

In Kenya, emergency nursing education is available through a one-year program (Kenya Registered Accident and Emergency Nursing) at the Cicely McDonell College of Health Sciences [43]. A one-year program for emergency nursing education is available at Kenyatta National Hospital, as are ongoing courses regarding key topics in emergency care 2., 44..

Emergency medical services are growing and while EMS staff providing out of hospital care are increasingly being formalized and educated, gaps in knowledge and lack of standardization remain. Neither level of EMS provider (EMT, paramedic) is fully recognized as a healthcare provider and there is a need to develop a national licensing framework as well as regulations for emergency medical technicians and ambulance operators [29].

## Under development

Now that emergency medicine has been gazetted as a specialty, there has been activity around efforts to develop Masters of Medicine in Emergency Medicine for Kenyan physicians. Curricula are currently in varying stages at several universities, including Aga Khan University (and its affiliated hospital) in Nairobi, Jomo Kenyatta University of Agriculture and Technology (and Kenyatta National Hospital) in Nairobi, and Moi University (with Moi Teaching and Referral Hospital) in Eldoret.

## The future of emergency care in Kenya

Nationally and locally, there is increasing governmental and non-governmental interest in improving the quality of emergency care in Kenya. The constitutional right for citizens to access adequate emergency care has highlighted the need to close the gap between the supply and demand of emergency care. Emergency medical care is a mandatory component of UHC and serves as the keystone to addressing the triple burden of disease. Devolution, however, has created challenges across a fractured landscape. There is also a need to develop a common reference point nationally for what emergency care is and will be in Kenya. Expanding and strengthening policies and the standard of emergency medical care will improve targets for institutional improvement.

## Universal health coverage

The goals of UHC underscore the importance of emergency medical care; aiming to provide access to necessary health services at any time and without undue financial cost. In effect, adequate emergency care systems (from prehospital to tertiary care) can provide crucially important and potentially lifesaving care at all hours. Emergency medicine trained providers can diagnose and treat communicable disease, the complications arising from non-communicable disease, as well as injuries; a spectrum of skills and knowledge that address UHC in a comprehensive manner. Further, under the UHC model, individuals who truly cannot afford to pay for emergent health services still obtain care without care being denied or delayed.

## Addressing challenges of devolution

Given that 47 semi-autonomous counties are each responsible for developing and coordinating local emergency care under the devolved healthcare model, there would be significant benefit from a national standard for emergency care in Kenya. Utilizing the WHO emergency care framework would seem to be most beneficial, given its role in integrating emergency care systems across levels of care and geography. Further, tailored national policies and standards for emergency care should be set forth by the Kenyan ministry of health, in close consultation with county governments and other non-governmental stakeholders.

Intra-county coordination around emergency care could occur both within and between Kenya's regional economic blocs. Synchronization of protocols and pooling of resources among neighboring counties has the potential to improve care and resource efficiency, particularly for time sensitive diagnoses and large multiple casualty events. The development of national standards (such as for pre-hospital care or the built environment of emergency wards) could raise the caliber of care locally and regionally. The Kenyan government should also take a leading role in coordinating a nationwide medical emergency phone number, which would help to coordinate emergency medical response at the local level (this could function in a manner similar to the network of regional poison centers in the United States that are unified under a single national phone number).

## Human resources and education

A stepwise approach to addressing the existing gaps in care can be redressed through expanding the pool of workers while also raising the level of provider skills and knowledge at all levels of care. However, gains could still be made by empowering the existing human resources that work in emergency care. This challenge is not unique to Kenya or even LMICs but does highlight the near-constant loss of institutional and emergency care knowledge as providers often move to other career opportunities or medical specialties. As noted above, many educational efforts in Kenya are through short courses, and short-term gains can quickly be lost through attrition as providers move on to another rotation, hospital, or career opportunity. There is a need to develop new methods of emergency medicine education and training that lie beyond brief short courses but are less burdensome than rigorous MMed training.

Multi-faceted strategies could include identifying and designating certain nurses, medical officers, and physicians as emergency ward staff, while also providing incentives and making other efforts to retain staff who have been trained in emergency care and/or have significant emergency work experience. Such changes should aim to reduce emergency ward staff turnover and frequent knowledge losses. Funding for emergency medical staff is crucial, and broad solutions are more difficult in the post-devolution model. Within counties and local hospitals, there is a need to identify and cultivate champions for emergency medicine, leaders who can develop viable career pathways and educate others to the value of quality emergency care.

The Kenyan post-graduate medical education system will hopefully be recruiting and (in short order graduating) its first cohorts of locally trained emergency medicine physicians; these individuals will step into roles as local and national leaders in emergency medicine. They will be responsible for building capacity at multiple levels and will likely need to tactfully guide institutional change. Subsequently, as has been seen in other African nations, many of these early graduates will form the foundation of the emergency medicine faculty. Hopefully undergraduate medical student curricula for emergency medicine will follow, as such work fosters students' interest in emergency medicine as a career path 45., 46..

Collaboration around specialty training among Kenyan universities and other regional partners is not a novel approach. The College of Surgeons of East, Central and Southern Africa (COSECSA) model for surgical training and education is based on utilizing accredited educators in certified health facilities with both in-person and online educational components [47]. Such a collaboration could be sought for physicians training in emergency medicine and could lend numerous potential benefits including shared resources, expanded capacity, professionalization and networking, and improved opportunities based on the experiences of others.

## Conclusion

Kenya has made advances in the care for emergent conditions in the past decade: emergency medicine is now a recognized specialty and emergency care is a governmental priority. Yet, gaps remain for the improvement of care and education, particularly given the challenges of devolution. As several Kenyan universities develop and expand the opportunities for specialized training, emergency medicine will hopefully be a blossoming field with growing demand and increasing quality. The ability to provide quality emergency care is a key to attaining universal health coverage, and to do so emergency care providers in Kenya must raise the standard of emergency care and education through innovative solutions that will improve care around the world.

## Funding

This research did not receive any grant from funding agencies in the public, commercial, or not-for-profit sectors.

## Dissemination of results

This work has been shared with the Emergency Medicine Kenya Foundation and will be further disseminated with relevant stakeholders at the local and national level within Kenya.

## Author contributions

Authors contributed as follows to the conception, research, drafting and revising the work critically for important intellectual content: JAL contributed 60%, BWW and ARA 10% each, and GW, JTS, NN and RK contributed 5% each. All authors approved the version to be published and agreed to be accountable for all aspects of the work.

## Declaration of competing interest

The authors declare no conflicts of interest.
